# Density of wild honey bee, *Apis mellifera*, colonies worldwide

**DOI:** 10.1002/ece3.10609

**Published:** 2023-10-11

**Authors:** Oliver D. Visick, Francis L. W. Ratnieks

**Affiliations:** ^1^ Laboratory of Apiculture and Social Insects (LASI), School of Life Sciences University of Sussex Brighton UK

**Keywords:** *Apis mellifera*, beekeeping, biogeography, natural selection, population size, wild colony density

## Abstract

The western honey bee, *Apis mellifera*, lives worldwide in approximately 102 million managed hives but also wild throughout much of its native and introduced range. Despite the global importance of *A. mellifera* as a crop pollinator, wild colonies have received comparatively little attention in the scientific literature and basic information regarding their density and abundance is scattered. Here, we review 40 studies that have quantified wild colony density directly (*n* = 33) or indirectly using genetic markers (*n* = 7) and analyse data from 41 locations worldwide to identify factors that influence wild colony density. We also compare the density of wild and managed colonies at a regional scale using data on managed colonies from the Food and Agriculture Organization (FAO). Wild colony densities varied from 0.1 to 24.2/km^2^ and were significantly lower in Europe (average of 0.26/km^2^) than in Northern America (1.4/km^2^), Oceania (4.4/km^2^), Latin America (6.7/km^2^) and Africa (6.8/km^2^). Regional differences were not significant after controlling for both temperature and survey area, suggesting that cooler climates and larger survey areas may be responsible for the low densities reported in Europe. Managed colony densities were 2.2/km^2^ in Asia, 1.2/km^2^ in Europe, 0.2/km^2^, in Northern America, 0.2/km^2^ in Oceania, 0.5/km^2^ in Latin America and 1/km^2^ in Africa. Wild colony densities exceeded those of managed colonies in all regions except Europe and Asia. Overall, there were estimated to be between two and three times as many wild colonies as managed worldwide. More wild colony surveys, particularly in Asia and South America, are needed to assess the relative density of wild and managed colonies at smaller spatial scales.

## Graphical abstract

Here, we provide a comprehensive review and analysis of all published literature regarding the density of wild honey bee colonies. Wild colony densities varied from 0.1 to 24.2 per km^2^ at 41 locations worldwide and were higher, on average, than managed colony densities in all regions except Europe.

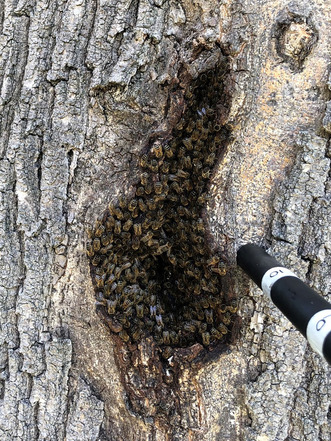

## 1 INTRODUCTION

**FIGURE 1:**
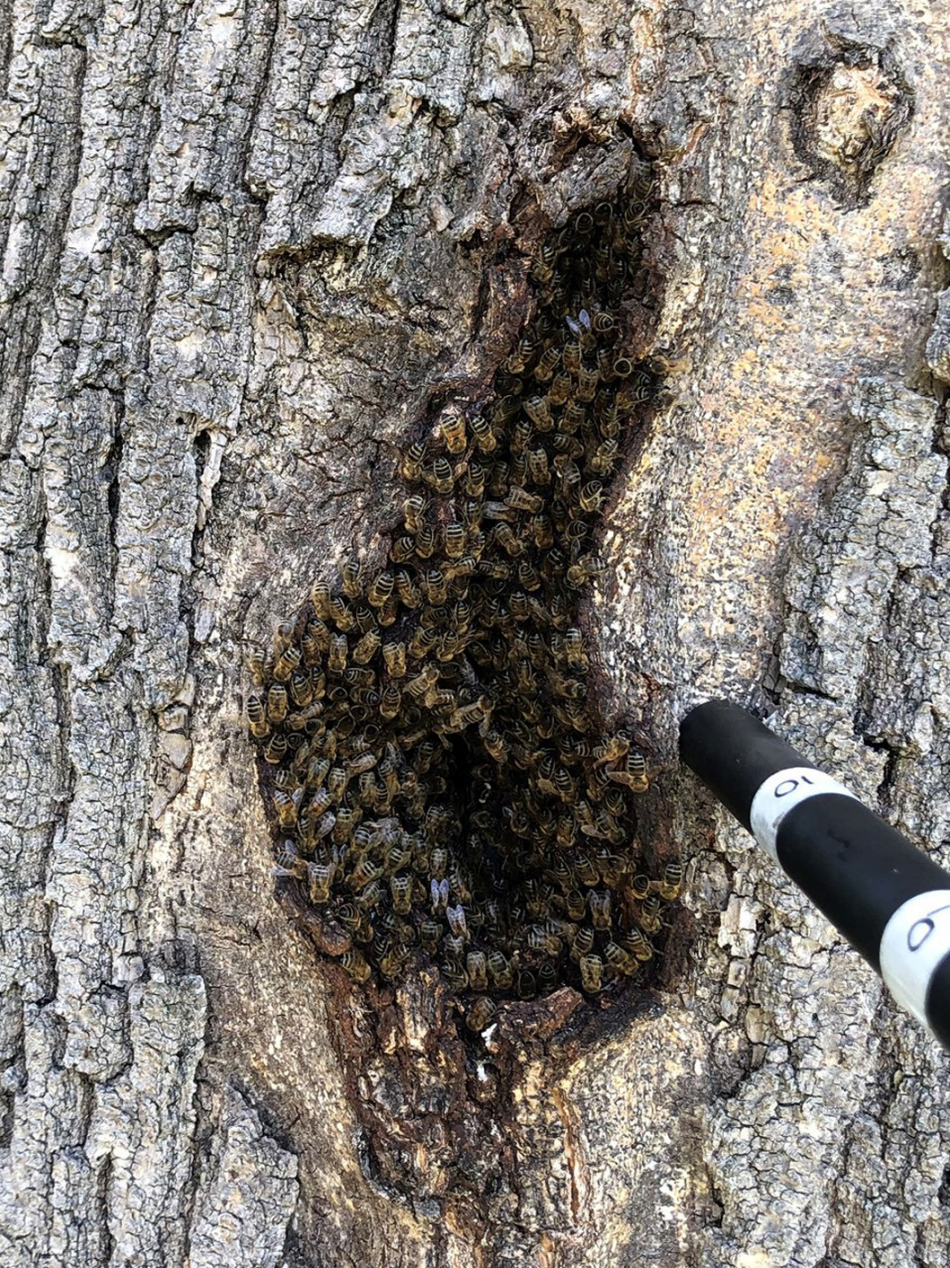
Wild honey bee, *Apis mellifera*, colony in a tree cavity in southern England.

Beekeeping with the western honey bee (*Apis mellifera* L.) dates back to ancient Egypt (Crane, 1999) and is now practiced on every continent except Antarctica, in both its native (Africa, Europe and Middle East) and introduced range (Americas, Asia and Oceania). In 2021, there were an estimated 102 million managed honey bee colonies worldwide (FAO, 2021), the majority being *A. mellifera*. *A. mellifera* pollinates approximately half of all globally important crops (Klein et al., 2007) and contributes over £100 billion to the global economy every year through pollination and honey production (Gallai et al., 2009). However, in addition to living in managed hives, *A. mellifera* colonies also live wild, typically nesting in cavities in trees (Figure 1) and buildings (Gambino et al., 1990; Saunders et al., 2021), but also in the ground and in rock crevices (Ratnieks et al., 1991) and occasionally not in a cavity (Boreham & Roubik, 1987; Saunders et al., 2021).

*Apis mellifera* is well‐studied in the contexts of general biology and beekeeping, but wild colonies are less studied (Seeley, 2019). For example, the first comprehensive study of the characteristics of natural nests (Seeley & Morse, 1976) was not made until several decades after the discovery of the dance language (von Frisch, 1937). Wild colonies are sometimes (Thompson, 2012), and incorrectly, viewed as a by‐product of beekeeping and have received comparatively little attention in the scientific literature (Kohl & Rutschmann, 2018). One important gap in our knowledge of *A. mellifera* is very basic: the density of wild colonies. Indeed, *A. mellifera* is listed as ‘data deficient’ on the ICUN Red List of Threatened Species (De la Rúa et al., 2014).

Wild *A. mellifera* colony densities were reviewed by Ratnieks et al. (1991) and found to range from 0.17 to 7.8/km^2^ at 9 locations worldwide (Africa: 1, Europe: 1, Latin America: 3, North America: 4). Two decades later, using an indirect method based on population genetics, Jaffé et al. (2010) estimated the densities of both wild and managed colonies combined at 25 locations across its native range (Africa: 10, Asia: 2, Europe: 13), which ranged from 0.8 to 10.2/km^2^. However, at most locations, the number of colonies detected using genetic markers could be accounted for by the estimated number of managed colonies, suggesting that wild colonies were rare or even absent in parts of its native range. However, indirect measures of colony density are subject to various limitations (reviewed by Utaipanon, Holmes, et al., 2019; Williamson et al., 2022), which make it difficult to detect wild colonies living at low densities in areas with many managed colonies (Kohl & Rutschmann, 2018).

Based on direct measures of wild colony density (Kohl & Rutschmann, 2018; Oleksa et al., 2013) and cavity density data, Requier et al. (2020) estimated the number of wild *A. mellifera* colonies that could be sustained in forests in a 4.6 million km^2^ area of Europe. Forests made up 1.4 million km^2^ (31%) of the study area and were estimated to contain approximately 80,000 wild colonies (0.057/km^2^ of forest). This is only 2% of the number of managed colonies reported in this region by the FAO (Requier et al., 2020), although the estimated number of wild colonies was based on lower bound estimates of colony density and did not account for wild colonies present in habitats other than forests (Requier et al., 2020). Therefore, the actual number of wild colonies present in Europe is likely much higher and probably represents an important component of total colony numbers.

Here, we review all studies that have measured the density of wild *A. mellifera* colonies with a focus on studies that used direct surveys to count colonies in defined areas. In the 30 years since the review by Ratnieks et al. (1991), there has been an upsurge in interest in pollinators and the number of wild colony surveys reported in the literature has increased three‐fold (see Section 4). *A. mellifera* is economically important and occurs worldwide and there are several reasons why information on the density of wild colonies is important, including the conservation of native subspecies (reviewed by Requier et al., 2019) and as sources of genetic variation in beekeeping (see below). Therefore, a comprehensive review is timely. In addition, we further analyse the data set to investigate factors that influence wild *A. mellifera* colony density. We also compare the density of wild and managed colonies at a regional scale using data on managed colonies from the FAO and make a tentative estimate for the number of wild *A. mellifera* colonies worldwide.

## 2 WHY IS WILD *A. MELLIFERA* COLONY DENSITY IMPORTANT?

Information on the density of wild *A. mellifera* colonies, in both its native and introduced ranges, is of both ecological and economic importance. For instance, measures of wild colony density in agricultural areas, in combination with information on foraging distances (Couvillon et al., 2014), could be used to assess their potential contribution to crop pollination. Indirect measures of wild colony density have been lower in agricultural areas compared to natural habitats (Hinson et al., 2015; Jaffé et al., 2010), possibly due to a lack of suitable nesting sites in the former (Oleksa et al., 2013). Therefore, the contribution of wild colonies to crop pollination in large‐scale farming operations is probably low compared to managed colonies which can be placed at very high densities for short periods of time, such as 2 per acre (500/km^2^) for almond pollination (Mader et al., 2010) and 6.7 per acre (1650/km^2^) for onion pollination (literature average, Delaplane & Mayer, 2000).

Measures of colony density can also be used to address concerns regarding the impact that both managed and wild colonies of *A. mellifera* have in their introduced range. Proposed threats include competition with native pollinators for floral resources (Paini, 2004), competition with native vertebrates for nest sites (Oldroyd et al., 1994; Pacífico et al., 2020), pollination of exotic weeds (Goulson & Derwent, 2004) and the introduction of exotic pests and disease (Taylor et al., 2007). In Australia, measures of colony density have been used to determine whether wild colonies pose a significant threat to native ecosystems and, in extreme cases, whether eradication would be possible (Hinson et al., 2015; Oldroyd, 1998; Oldroyd et al., 1994).

Repeated surveys can also be used to monitor changes in wild colony density over time. For example, in the Arnot Forest in western New York State, three surveys spanning over 30 years (1978, 2002 and 2011) showed that the introduction of the ectoparasitic mite, *Varroa destructor*, in the 1990s had no long‐term effect on the density of wild *A. mellifera* colonies living in the area (Seeley, 2007; Seeley et al., 2015; Visscher & Seeley, 1982) even though *Varroa* became established between the first and second surveys. Similarly, in The Welder Wildlife Refuge, Texas, two surveys conducted both during (1991–2001) and after (2013) the invasion of Africanised bees showed that wild colony densities remained high over a decade after the invasion (5.4/km^2^) and that wild colonies retained a stable mixture of European‐ and African‐derived genetics. These studies are important in showing that wild populations of *A. mellifera* can remain stable over long periods despite significant changes in the bees themselves or their pests.

Importantly, the role of natural selection on colony survival will be affected by the number of wild colonies present in an area. Many types of disease management, including the use of chemicals to kill *Varroa* mites, play an important role in the survival of managed colonies (van Dooremalen et al., 2012), but will likely reduce the effect of natural selection for disease resistance (Neumann & Blacquiere, 2016). Several wild and unmanaged populations of *A. mellifera* have been shown to possess natural mechanisms that reduce *Varroa* population growth (Mondet et al., 2020) including short post‐capping durations (Le Conte et al., 2007; Oddie et al., 2018), cell recapping (Hawkins & Martin, 2021; Oddie & Dahle, 2021) and *Varroa*‐sensitive hygiene (Harris, 2007; Panziera et al., 2017). In a typical population of *A. mellifera*, where wild and managed colonies can interbreed over long distances (De la Rúa et al., 2013), heritable traits that confer long‐term resistance to *Varroa* will likely increase in frequency more rapidly when a greater proportion of colonies are wild and exposed to natural selection (Requier et al., 2019; Youngsteadt et al., 2015). These traits are also present in managed colonies at low frequencies and can be increased via artificial selection and queen rearing (Bigio et al., 2014; Büchler et al., 2010; Pérez‐Sato et al., 2009; Rinderer et al., 2010).

## 3 METHODS

### 3.1 Choice of studies

Here, we review 55 reports of wild honey bee colony density from a total of 40 studies published between 1971 and 2022, although the actual data from Galton (1971) originates from the late 1600s (Seeley, 2019). 33 reports come from regions where *A. mellifera* is introduced (Australia: 16, Latin America: 7, USA: 10), versus 22 from within *A. mellifera's* native range (Africa: 13, Europe: 9). Interestingly, there have been no reports of established wild populations of *A. mellifera* in eastern or southern Asia despite their increasing use in commercial beekeeping. This is suggested to be due to the parasites and diseases of other species of honey bee present in these regions (e.g. *Apis cerana*; Oldroyd & Nanork, 2009; see Section 5).

Direct measures of wild colony density (*n* = 35, Table 1) were made using one or more of the following methods. Direct searches were the most common (*n* = 19) and involved looking for bees going in and out of nesting cavities. Nine surveys were made using bee‐lining, which involves following marked bees back to their nest by recording the direction they depart from a food source and the time it takes them to return (Seeley, 2016). Four surveys were made using local knowledge, which involved communications with local residents, landowners and African honey hunters (Kajobe & Roubik, 2006; Schneider & Blyther, 1988). Four surveys were made using data from either forest beekeeping in Russia (*n* = 1), in which honey from wild colonies living in trees is harvested (Seeley, 2019) or colony removal records from urban areas (*n* = 3).

**TABLE 1:** Thirty‐six reports from 33 studies that have quantified wild *A. mellifera* colony density directly.

Country	Location	Latitude	Longitude	Habitat	Survey method(s)	Main cavity type	No. plots/sites	No. colonies	Survey area (km^2^)	Colonies/km^2^	Reference
Russia (N)	Morozov Estate	56.3*	44.0*	Temperate forest	Colony records	Tree hollows	4	3–50	10–88	0.17–0.96	Galton (1971)^a^
Brazil (I)	Goiás & Mato Grosso	−18.5*	−52.4*	Woodland	Direct search^b^	Tree hollows	15	8	1.80	4.4	Kerr (1971)^a^
Ivory Coast (N)	Lamto Savannah	6.6*	−5.3*	Dry forest	Direct search^b^	Tree hollows	94	3	0.30^c^	10	Darchen (1972)^d^
USA (I)	Cottonwood, AZ	34.7	−112.0	Semi–desert canyon	Direct search	Rocks crevices	–	9–16	3.14	2.9–5.1	Taber (1979)
USA (I)	Arnot Forest, NY	42.3	−76.7	Temperate forest	Bee–lining	Trees hollows	–	9	8.5	1.06	Visscher and Seeley (1982)
Panama (I)	Panama Canal	9.1*	−79.7*	Mixed	Colony records	Man–made	–	60–105^e^	50	1.2–2.1	Boreham and Roubik (1987)
Botswana (N)	Okavango	−19.6	23.4	Semi–desert	Local Knowledge	Trees hollows	–	47	6	7.8	Schneider and Blyther (1988)
USA (I)	Santa Cruz Island, CA	34.0	−119.7	Arid Island	Bee–lining	Rocks crevices	–	58^f^	230	0.25	Wenner (1989)
USA (I)	Oswego, NY	43.5	−76.5	Urban/suburban	Local knowledge	Man–made	–	11	4.2	2.3	Morse et al. (1990)
Mexico (I)	Tapachula	14.9	−92.3	Agricultural land	Search & local	Tree hollows	3	5–13	1–2.1	5–9	Ratnieks et al. (1991)
Australia (I)	Wyperfeld NP, Vic	−35.6	141.9	Riparian woodland	Direct search	Tree hollows	7	27	0.35^c^	77.1	Oldroyd et al. (1994)
Costa Rica (I)	Belén	10.4	−85.6	Patchy tropical forest	Bee–lining, search & local	Tree hollows	–	38	12.6	3.02	Danka et al. (1994)
Australia (I)	South Australia	−35.6*	139.1*	Conservation parks	Direct search^b^	–	5	1–60	0.09–20	0.11–40	Paton, DC, Jansen, L & Oliver, D (unpublished)^g^
Botswana (N)	Okavango	−19.6	23.4	Semi–desert	Search & local	Tree hollows	–	81	19.3	4.2	McNally and Schneider (1996)^h^
Australia (I)	Wyperfeld NP, Vic	−35.6	141.9	Riparian woodland	Direct search	Tree hollows	5	10–37	0.25	40–148	Oldroyd et al. (1997)
Australia (I)	Box‐ironbark Forest, Vic	−36.1*	146.6*	Box–ironbark Forest	Direct search	Tree hollows	35	1	0.35^c^	2.9	Goodman and Hepworth (2004)
Australia (I)	Goulburn Valley, Vic	−36.4*	145.4*	Riparian woodland	Direct search	Tree hollows	30	84	0.91^c^	92	Goodman and Hepworth (2004)
USA (I)	Welder Wildlife Refuge, TX	28.1	−97.4	Coastal prairie	Direct search	Tree hollows	–	10–79^i^	6.25	1.6–12.64	Baum et al. (2005)
Uganda (N)	Bwindi Impenetrable NP	−0.6	29.7	Montane forest	Local knowledge^j^	Tree hollows	87	20	1.74	12	Kajobe and Roubik (2006)
USA (I)	Arnot Forest, NY	42.3	−76.7	Temperate forest	Bee–lining	Tree hollows	–	8	8.5	0.94	Seeley (2007)
USA (I)	Tucson, AZ	32.2	−111.0	Urban/suburban	Colony records	Man–made	–	323–1035	924.1^k^	0.35–1.12	Baum et al. (2008)
Poland (N)	Northern Poland	53.7*	19.9*	Rural avenues	Direct search	Tree hollows	170	45	458.1^l^	0.1	Oleksa et al. (2013)
USA (I)	Arnot Forest, NY	42.3	−76.7	Temperate forest	Bee–lining	Tree hollows	–	9	8.5	1.06	Seeley et al. (2015)
USA (I)	Welder Wildlife Refuge, TX	28.1	−97.4	Coastal prairie	Direct search	Tree hollows	–	28	5.14	5.4	Rangel et al. (2016)
South Africa (N)	Cape Point	−34.4*	18.5*	Fynbos (heathland)	Bee–lining & search	Rock crevices	–	59	50.4^m^	1.17	Tribe et al. (2017)
Germany (N)	Hainich NP	51.1	10.4	Temperate forest	Bee–lining	Tree hollows	–	4–6^n^	30.6–43.2	0.13	Kohl and Rutschmann (2018)
Germany (N)	Swabian Alb	48.4	9.5	Temperate forest	Direct search^o^	Tree hollows	–	7	61.3	0.11	Kohl and Rutschmann (2018)
USA (I)	Shindagin Hollow	42.3	−76.3	Temperate forest	Bee–lining	Tree hollows	–	5	5.18	0.97	Seeley and Radcliffe (2018)
Zambia (N)	Lusaka	−14.2	30.2	Miombo/mopane	Direct search	Tree hollows	2	29	1.2	24.2	Coppinger et al. (2019)
Scotland (N)	Cawdor Wood	57.5	−3.9	Woodland	Bee–lining	Tree hollows	–	4	31.1	0.13	Seeley and Chilcott (2020)
Serbia (N)	Belgrade	44.8	20.5	Urban/suburban	Colony records	Man–made	–	460^p^	224	2.05	Bila Dubaić et al. (2021)
Spain (N)	Xinzo de Limia	42.1	−7.7	Agricultural land	Direct search^q^	Man–made	–	23–29	136	0.17–0.22	Rutschmann et al. (2022)
Australia (I)	Albury, NSW	−35.5*	147.8*	Patchy woodland	Direct search^r^	Man–made	10	36	0.53^c^	68.6	Cunningham et al. (2022)
Germany (N)	Coburg & Lichtenfels	50.2*	11*	Temperate forest	Direct search^o^	Tree hollows	–	0–21	22.1–59.1	0–0.36	Kohl et al. (2022)
Germany (N)	Swabian Alb	48.4	9.5	Temperate forest	Direct search^o^	Tree hollows	–	0–20	48.6–116.9	0–0.21	Kohl et al. (2022)
Australia (I)	Adelaide, SA	−35.0	138.6	Arboretum/urban	Direct search	Tree hollows	–	31–81	1.34–7.65	23.1–10.59	Williamson et al. (2022)

*Note*: The letter in column 1 corresponds with whether each country is in *A. mellifera's* native (N) or introduced (I) range. A dash (–) in column 8 indicates that a contiguous area was surveyed. An asterisk (*) next to a coordinate in column 3 or 4 indicates that it is was either approximated or averaged between sites.

^a^ From Ratnieks et al. (1991).

^b^ Survey method not reported (assumed to be direct search).

^c^ Densities measured in areas <1 km^2^ were included in the table for completeness but omitted from analyses.

^d^ From Kajobe and Roubik (2006).

^e^ Range of colonies removed over 4‐year period. Ratnieks et al. (1991) reported both colonies and swarms.

^f^ Exact number of colonies not reported. Calculated from colony density and area surveyed.

^g^ From Paton (1996).

^h^ Combined with data from Schneider and Blyther (1988).

^i^ From Rangel et al. (2016).

^j^ Colonies located by guides and indigenous honey hunters.

^k^ Survey area calculated from reported colony density and numbers.

^l^ Forty‐five colonies located on 142 km of rural avenues. Colony density estimated via avenue density.

^m^ Searched 65% of the Cape Point section of the Table Mountain National Park (77.5 km^2^).

^n^ Nest not actually located (location inferred by bee‐lining).

^o^ Only searched tree hollows that were previously occupied by black woodpeckers.

^p^ Four hundred sixty colonies identified during 7‐year study period. Only c. 20% were continuously occupied during study period.

^q^ Only searched concrete power poles.

^r^ Only searched nest boxes.

Indirect measures of wild colony density (*n* = 19, Table 2) were made by taking samples of honey bees from either the worker progeny of queens mated in an area of interest (Arundel et al., 2014; Jaffé et al., 2010; Moritz et al., 2007) or, more commonly, from drones trapped at drone congregation areas (DCAs; Arundel et al., 2013; Hinson et al., 2015; Jaffé et al., 2010; Moritz et al., 2007, 2008, 2013; Utaipanon, Holmes, et al., 2019; Utaipanon, Holmes, et al., 2021; Utaipanon, Schaerf, et al., 2021). The number of colonies within a given radius of the mating apiary or DCA is then inferred by the number of unique genotypes present in the sample of worker progeny or trapped drones, respectively. Indirect measures of wild colony density were only included if they excluded the effect of managed colonies on the total number of colonies detected. For example, Jaffé et al. (2010), Arundel et al. (2014), Hinson et al. (2015) and Utaipanon, Schaerf, and Oldroyd (2019) sampled sites with little or no managed colonies within drone flight distance.

**TABLE 2:** Nineteen reports from seven studies that have quantified wild *A. mellifera* colony density indirectly using genetic markers.

Country	Location	Latitude	Longitude	Habitat	Sampling method	Drones genotyped	No. sites	No. colonies	Survey area (km^2^)	Colonies/km^2^	Reference
South Africa (N)	Gauteng^a^	−25.9	28.6	Nature reserve	Drone trapping	96–127	3	32–44	2.5	12.8–17.6	Moritz et al. (2007)
South Africa (N)	Tswalu game reserve^a^	−27.2	22.4	Nature reserve	Drone trapping	49	–	26.5	2.5	10.6	Moritz et al. (2008)
South Africa (N)	Gauteng^a^	−25.9	28.6	Nature reserve	Drone trapping	96–191	3	23–37	2.5	9.2–14.8	Jaffé et al. (2010)
South Africa (N)	Tswalu game reserve^a^	−27.2	22.4	Nature reserve	Drone trapping	148	–	29	2.5	11.6	Jaffé et al. (2010)
South Africa (N)	Jonkershoek^a^	−34.0	18.9	Nature reserve	Drone trapping	96	–	40	2.5	16	Jaffé et al. (2010)
South Africa (N)	Pietermaritzburg^a^	−29.6	30.5	Nature reserve	Drone trapping	96	–	52	2.5	20.8	Jaffé et al. (2010)
Sudan (N)	Al‐faw^a^	14.2	34.3	Agricultural land	Worker progeny	–	–	23	4.5	5.1	Jaffé et al. (2010)
Mexico (I)	Chiapas N.^b^	16.7*	−92.4*	Agricultural land	Drone trapping	89–96	5	38–52	2.5	20.8–15.2	Moritz et al. (2013)
Mexico (I)	Chiapas S.^b^	14.9*	−92.5*	Mangrove & mango plantation	Drone trapping	89–183	5	34–43	2.5	13.6–17.2	Moritz et al. (2013)
Mexico (I)	Yucatan^b^	21.1*	−88.9*	Deciduous forest	Drone trapping	92	2	37–42	2.5	14.8–16.8	Moritz et al. (2013)
Australia (I)	Wyperfeld NP & Yaapeet, Vic^c^	−35.6*	142.0*	Mallee^e^	Worker progeny	–	2	25–29	4.5^f^	5.6–6.4	Arundel et al. (2014)
Australia (I)	Puckapunyal N. & S., Vic^c^	−36.96*	144.9*	Box ironbark forest	Worker progeny	–	2	3–11	4.5^f^	0.67–2.4	Arundel et al. (2014)
Australia (I)	Puckapunyal E. & Dookie, Vic^c^	−36.6*	145.4*	Box ironbark forest^e^	Worker progeny	–	2	14–18	4.5^f^	3.1–4	Arundel et al. (2014)
Australia (I)	Marysville & Eildon, Vic	−37.4*	145.8*	Sclerophyll forest^e^	Worker progeny	–	2	13–15	4.5^f^	2.9–3.3	Arundel et al. (2014)
Australia (I)	Barrington Tops, NSW^d^	−32.2*	151.7*	Sclerophyll forest^e^	Drone trapping	70–123	4	14–23.5	2.5^g^	5.6–9.4	Hinson et al. (2015)
Australia (I)	Weddin Shire, NSW^d^	−33.6*	148.1*	Woodland^e^	Drone trapping	14–241	4	6–49	2.5^g^	2.4–19.6	Hinson et al. (2015)
Australia (I)	Wimmera, Vic^d^	−35.6*	142.1*	Mallee^e^	Drone trapping	62–278	4	18–37.5	2.5^g^	7.2–15	Hinson et al. (2015)
Australia (I)	Grong Grong, NSW	−34.8	146.7	Mixed farming	Drone trapping	24–251	15	236	86.5^h^	2.73	Utaipanon, Schaerf, and Oldroyd (2019)
Australia (I)	Currawarna, NSW	−35.0	147.1	Mixed farming	Drone trapping	22–216	15	265	86.5^h^	3.06	Utaipanon, Schaerf, and Oldroyd (2019)

*Note*: All indirect measures have been made over the last 15 years and are mainly limited to the Southern hemisphere. The letter in column 1 corresponds with whether each country is in *A. mellifera's* native (N) or introduced (I) range. An asterisk (*) next to a coordinate in column 3 or 4 indicates that it is was either approximated or averaged between sites.

^a^ No managed colonies within a 2.5–3 km radius of the sampling site.

^b^ No difference in number of colonies detected in areas with high and low beekeeping.

^c^ Beekeeping not practiced in study location for 40–50 years.

^d^ Beekeeping either prohibited from study locations or absent during time of sampling.

^e^ Survey location consisted of paired disturbed and undisturbed sites.

^f^ Original survey area and density calculated using agent‐based model from Arundel et al. (2012).

^g^ Original survey area and density calculated using agent‐based model from Arundel et al. (2013).

^h^ Survey area calculated at two 7 km transects with typical drone flight range of 3.75 km.

### 3.2 Area surveyed

The area over which wild colonies were located (direct) or detected (indirect) was, in most cases, explicitly stated by the authors in their calculation of wild colony density. Survey areas were sometimes given as the total area of multiple plots of a standard size (<0.05 km^2^ each; Darchen, 1972; Goodman & Hepworth, 2004; Kajobe & Roubik, 2006; Oldroyd et al., 1994, 1997) or the area of a circle with a given radius (Danka et al., 1994; Morse et al., 1990; Seeley & Radcliffe, 2018; Seeley & Chilcott, 2020; Taber, 1979), but in most cases the origin of survey areas was not given. In cases where survey areas were not explicitly stated, they were estimated by either dividing the number of colonies by the reported density (Baum et al., 2008; McNally & Schneider, 1996) or by using other information provided by the authors. For example, survey areas in Oleksa et al. (2013) and Kohl et al. (2022) were calculated using the density of rural avenues and cavity trees, respectively.

Areas surveyed using indirect measures were assumed to be either 2.5 km^2^ (drone trapping) or 4.5 km^2^ (worker progeny), based on the assumption that drones mate at a median distance of 900 m from their colony (Taylor & Rowell, 1988) and that queens mate over an area approximately 1.8 times as large (Jaffé et al., 2010). An exception to this is Utaipanon, Schaerf, and Oldroyd (2019), who measured their own drone mating distances and found that drones caught along two 7‐km transects in New South Wales were sampled from a much larger area of 86.5 km^2^. Honey bee mating distances can vary significantly (Jensen et al., 2005) and even a small increase in distance can have a large effect on the resulting area (Utaipanon, Schaerf, & Oldroyd, 2019).

### 3.3 How multiple surveys at a location were combined

Data from each survey location were combined to produce a single value for wild colony density. In some locations, data were combined over multiple years (Baum et al., 2005, 2008; Bila Dubaić et al., 2021; Boreham & Roubik, 1987; Kohl et al., 2022; Paton, 1996; Rutschmann et al., 2022; Taber, 1979) or across multiple sites or plots within a wider general location (Darchen, 1972; Galton, 1971; Goodman & Hepworth, 2004; Ilyasov et al., 2015; Kajobe & Roubik, 2006; Kerr, 1971; Oldroyd et al., 1994, 1997; Oleksa et al., 2013; Paton, 1996; Ratnieks et al., 1991). In the former, the mean number of wild colonies located each year was divided by the survey area, which was assumed to remain constant, as some colonies reported in each year would have been the same colonies as the previous year. For instance, using colony removal records, Baum et al. (2008) inferred the location of wild colonies in a 900 km^2^ area of Tucson, Arizona, during the invasion of Africanised bees from 1994 to 2001. The mean number of colonies located each year was 644.7, which produced a combined density of 0.7/km^2^. In cases where survey area did not remain constant (Kohl et al., 2022; Williamson et al., 2022), only data from the most recent survey were used to calculate density.

To combine data collected at different sites or plots within a survey location the total number of colonies located (direct) or detected (indirect) was divided by the total survey area. For example, Ratnieks et al. (1991) located 27 colonies in three sites near Tapachula, Mexico, with a total area of 4.1 km^2^, which resulted in a combined density of 6.6/km^2^. The same method was used to calculate regional densities of managed colonies using the total landmass (km^2^) of countries where FAO data are available (*n* = 117).

### 3.4 Criteria that excluded a study

**FIGURE 2:**
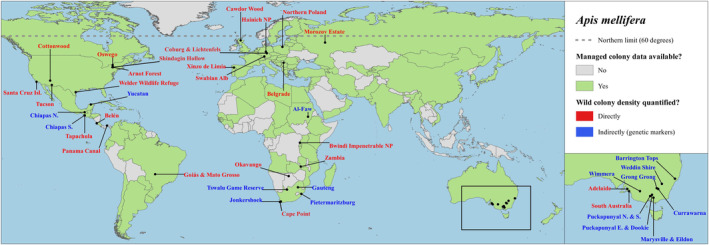
Forty‐one locations worldwide where wild *Apis mellifera* colony density has been quantified directly (red) or indirectly via genetic markers (blue). The grey dashed line indicates the approximate northern limit of wild colonies in Europe (60 degrees), which is based on the distribution of lime and hazel trees (Requier et al., 2019). Countries in green (*n* = 117) have FAO data regarding the number of managed honey bee colonies. Map generated in QGIS (v3.16.11).

In cases where a location had been surveyed by different studies using the same survey method, only data from the most recent study were included in the analysis. This includes the Arnot Forest, Okavango, Welder Wildlife Refuge, Wyperfeld National Park (Wimmera), Gauteng and Tswalu Game Reserve (Figure 2). Studies that surveyed a total area of <1 km^2^ (Cunningham et al., 2022; Darchen, 1972; Goodman & Hepworth, 2004; Oldroyd et al., 1994, 1997) were not included in the analysis because these often produced unrepresentative high densities (>50/km^2^, Table 1) that were probably not representative of the surrounding habitats.

### 3.5 Statistical analyses

All analyses were performed in R (v4.3.1; R Core Team, 2023) and all plots were made using *ggplot2* (Wickham, 2016). We analysed data from a total of 41 locations where wild colony density had been quantified either directly (*n* = 25) or indirectly using genetic markers (*n* = 16; Table S1). Each survey location was allocated to one of five broad geographical regions (Africa, Europe, Latin America, Northern America, Oceania). The Americas were split into Northern (USA and Canada) and Latin America rather than North and South America because only one report of wild colony density has been made in South America (Kerr, 1971). Each location was also allocated to one of three categories of land use (disturbed, undisturbed, mixed) based on the level of human disturbance (similar to Arundel et al., 2014; Hinson et al., 2015). For instance, natural habitats, such as nature reserves and unmanaged woodland, were listed as undisturbed (*n* = 20), whereas agricultural land and urban areas were listed as disturbed (*n* = 12) and locations consisting of both were listed as mixed (*n* = 9; see Tables 1 and 2 for a full list of habitats).

Climate data were obtained in the form of raster datasets with a global coverage. The mean value of each variable was extracted from a 50 km radius around each survey location using the Zonal Statistics tool in QGIS (v3.16.11). Simulated monthly temperature (°C) and precipitation (mm) data, with a spatial resolution of 0.5 by 0.5 degrees, were obtained from the Climatic Research Unit Time‐Series (v4.04; Harris et al., 2020) and averaged from 1970 to 2020. Monthly net primary productivity (gC/m^3^/day) data, with a spatial resolution of 0.1 by 0.1 degrees, were obtained from NASA's terra MODIS satellite (product key: MOD17, v6.1; Running & Zhao, 2021) and averaged from 2001 to 2015.

### 3.6 Model selection

Generalised linear models (GLMs) with Gamma error distributions were used to test for the effect of region, land use, climate and survey area on density at the 41 locations. Models with and without a variable were compared using their Akaike information criterion (AIC), a measure of goodness of fit that penalises models with more variables. A lower AIC indicates that a model better fits the data, although a difference in AIC (ΔAIC) of <2 is considered non‐significant. Models with only one variable were compared with the null model, which only includes an intercept term. Tukey HSD tests were used to test for regional differences in density after controlling for other variables such as climate and survey area. Tukey tests were run using *multcomp* (Hothorn et al., 2008) and *p* values were adjusted using the Bonferroni method. Geometric means are given for density and area because they both occur on a logarithmic scale.

### 3.7 FAO data on managed colonies

FAO data on managed colonies were available for 117 countries (Africa: 24, Asia: 26, Europe: 33, Latin America: 22, Oceania: 10, Northern America: 2; Table S2; Figure 2). Only the most recent reports of managed colony numbers were used to calculate density. For most countries, these were made in 2021, except for Guadeloupe (1990), the Netherlands (1987), the United Kingdom (1987) and Belgium (2017). The FAO does not specify the species of honey bee that are managed in each country, and in particular, the proportion of managed colonies in southern and eastern Asia that are *A. cerana*. However, it is believed that, even in eastern Asia, most managed colonies are *A. mellifera* (Osterman et al., 2021). *A. cerana* is native only to Asia, but not including western Asia and had been introduced into New Guinea and Queensland, Australia (Koetz, 2013).

## 4 RESULTS

### 4.1 Regional variation in wild colony density

**FIGURE 3:**
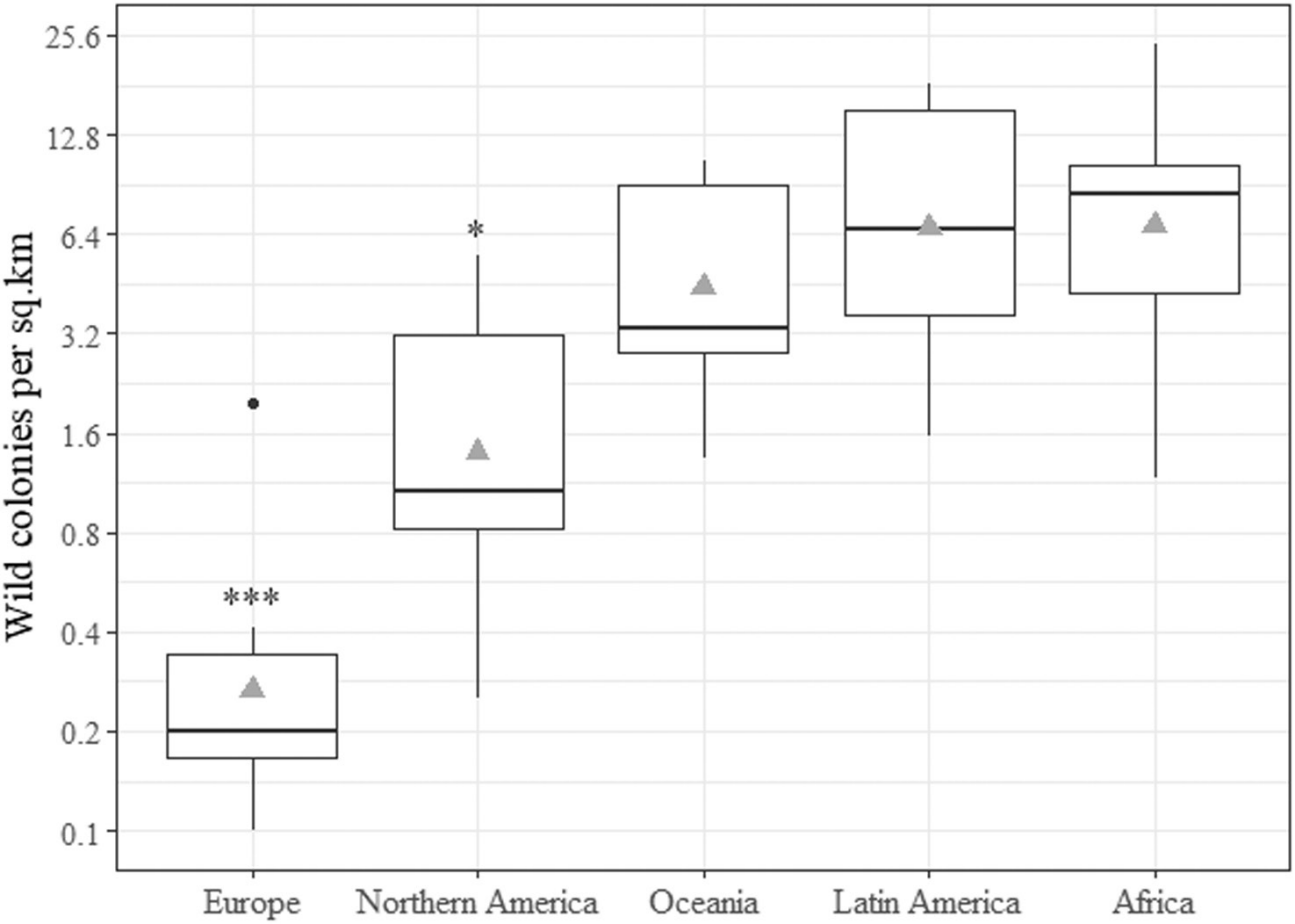
Regional variation in wild *Apis mellifera* colony density. Densities are plotted on a log scale because they vary over approximately 2.4 orders of magnitude. Mean regional densities (grey triangles) are 0.26/km^2^ in Europe (*n* = 8), 1.4/km^2^ in Northern America (*n* = 7), 4.4/km^2^ in Oceania (*n* = 10), 6.7/km^2^ in Latin America (*n* = 7) and 6.8/km^2^ in Africa (*n* = 9). Asterisks correspond with *p* values < .001 (***) and .05 (*). Boxes show the interquartile range, horizontal lines in boxes show the median and whiskers show the full range excluding outliers. The only outlier was a density of 2/km^2^ reported by Bila Dubaić et al. (2021) in Serbia (Europe).

Wild colony densities reported in the literature were highly variable, ranging from 0.1/km^2^ in Northern Poland to 148/km^2^ in Australia (Table 1). Densities in our sample of 41 locations worldwide (Figure 2), ranged from 0.1 to 24.2/km^2^ and fit a Gamma distribution with a geometric mean of 2.5/km^2^. Region had a significant effect on density (ΔAIC = 30.7). Densities reported in Europe (average of 0.26/km^2^) were significantly lower than Northern America (1.4/km^2^, *p* = .01), Oceania (4.4/km^2^, *p* < .001), Latin America (6.7/km^2^, *p* < .001) and Africa (8.4/km^2^, *p* < .001; Figure 3). Densities reported in Northern America were significantly lower than Africa (*p* = .022) and Latin America (*p* = .033) but not Oceania (*p* = .39). Land use had no effect on density (ΔAIC = −2.2).

### 4.2 Effect of climate

**FIGURE 4:**
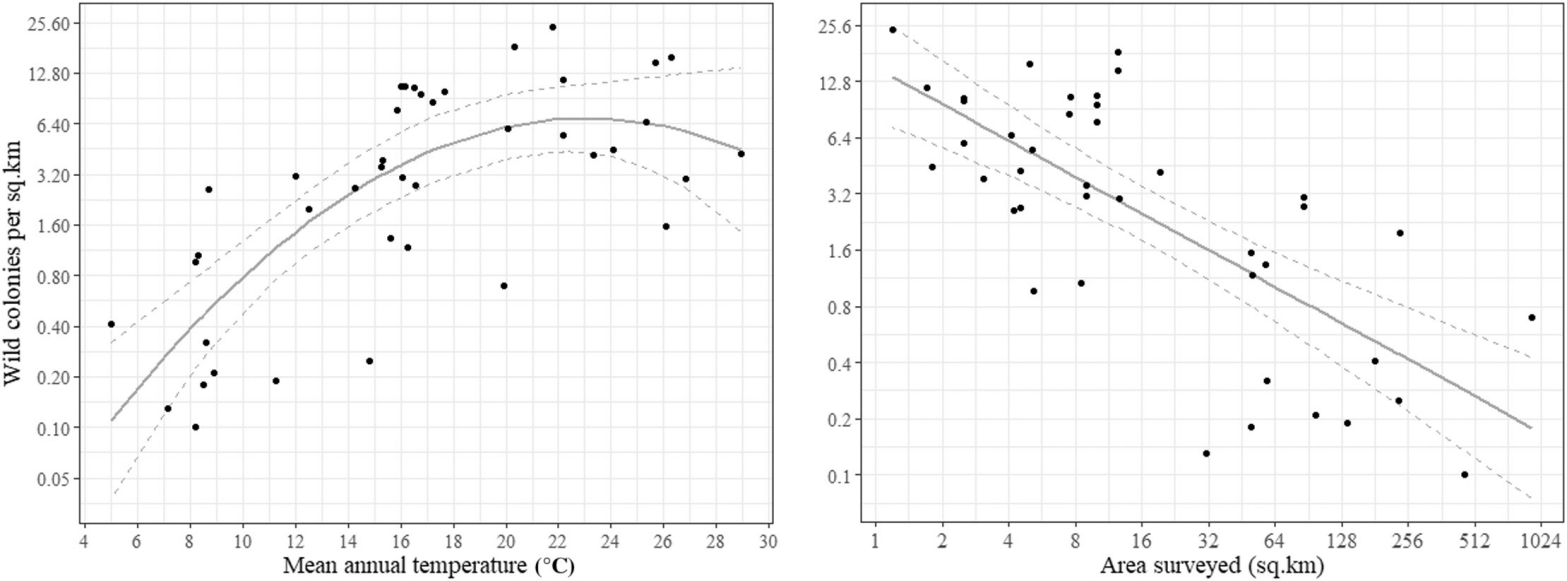
Effect of mean annual temperature (left) and survey area (right) on wild *Apis mellifera* colony density. Temperature had a significant quadratic component with densities decreasing at mean annual temperatures exceeding 23°C (*R* ^2^ = .55). Density had a negative correlation with survey area (*R* ^2^ = .53). Fit lines and 95% confidence intervals were calculated using a linear model and log‐transformed densities and areas.

There was a significant positive correlation between wild colony density and mean annual temperature (ΔAIC = 18.2). Temperature also had a significant quadratic component (ΔAIC = 9.9) with densities starting to decrease at mean annual temperatures exceeding 23°C (Figure 4). This model was not a better fit when other variables were included, such as mean monthly rainfall (ΔAIC = −1.5) and net primary productivity (ΔAIC = 1.6), although the latter was borderline significant. Region still had a significant effect on density after controlling for temperature (ΔAIC = 5.6), with densities remaining significantly lower in Europe (*p* < .05) but not Northern America (*p* > .1), compared to the other three regions.

### 4.3 Effect of survey area

Survey area varied considerably over nearly 3 orders of magnitude, even after excluding studies that surveyed <1 km (see methods), ranging from 1.2 km^2^ in Zambia to 924 km^2^ in Tucson, Arizona. Survey area had a significant negative correlation with density (ΔAIC = 24.3; Figure 4) and was significantly larger in Europe (average of 111 km^2^) and Northern America (18 km^2^), compared to Latin America (8.7 km^2^, *p* < .01) and Africa (4.7 km^2^, *p* < .01) but not Oceania (16.2 km^2^, *p* > .1). Region still had a significant effect on density after controlling for survey area (ΔAIC = 13.2). However, region no longer affected density after controlling for both survey area and mean annual temperature (ΔAIC = −1.2).

### 4.4 FAO managed colony numbers

**FIGURE 5:**
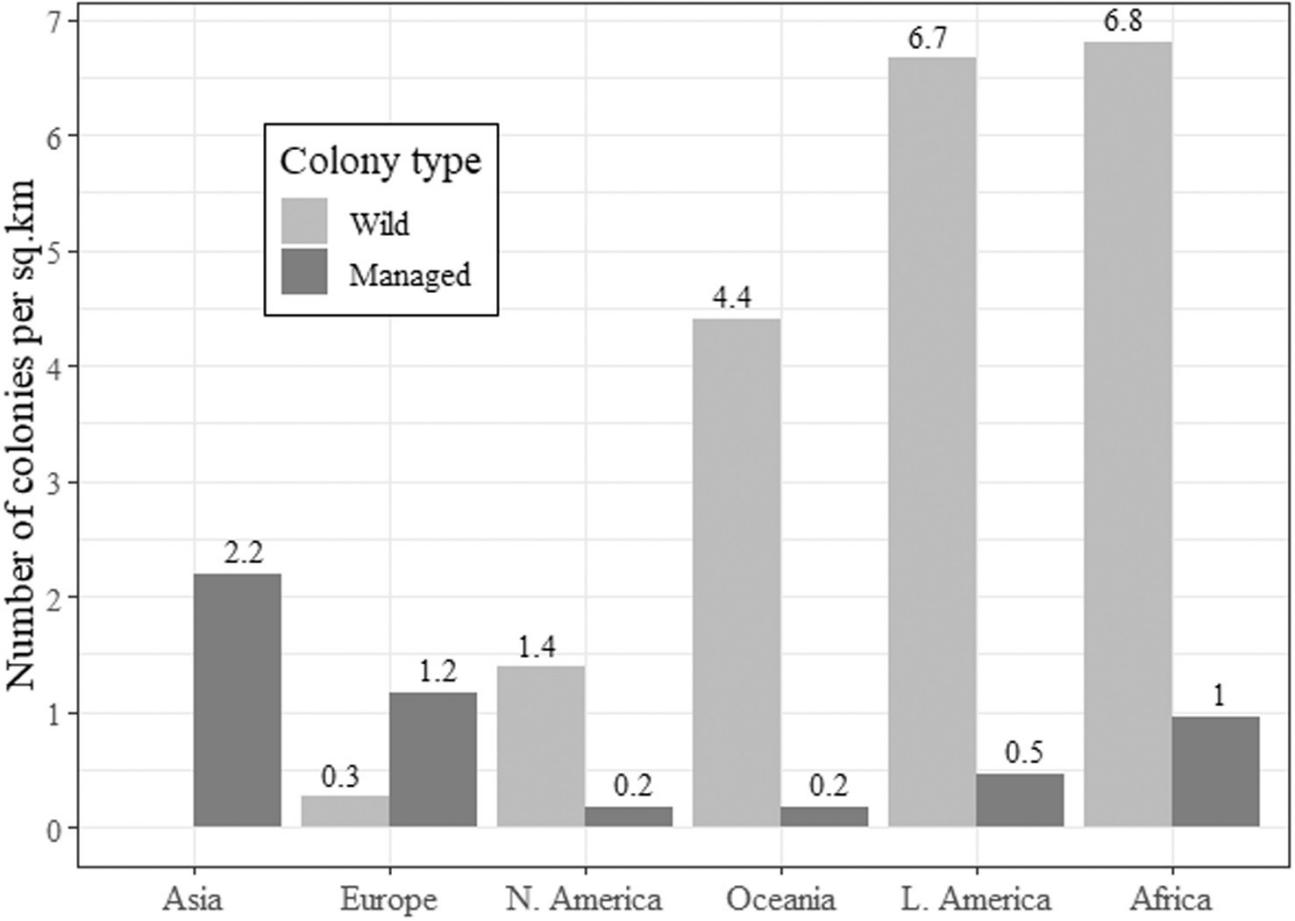
Density of wild and managed *A. mellifera* colonies. Wild colony densities are the mean densities plotted in Figure 3. Managed colony densities were calculated using the most recent data from the FAO (2021) and the landmass of countries where data are available (*n* = 117). Wild colony densities exceed those of managed colonies in all regions except Europe. There have been no reports of wild colony density in Asia.

Using recent data from the FAO, the number and density of managed colonies were calculated to be 1.4 million (0.18/km^2^) in Oceania, 3.4 million (0.18/km^2^) in Northern America, 8.2 million (0.47/km^2^) in Latin America, 18.2 million in Africa (0.96/km^2^), 25.4 million (1.2/km^2^) in Europe and 45.3 million (2.2/km^2^) in Asia. Managed colony densities were lower than mean wild colony densities in all regions except Europe and Asia (Figure 5).

## 5 DISCUSSION

### 5.1 Regional variation in wild colony density

Our results show that wild *A. mellifera* colony densities vary over approximately 2.4 orders of magnitude from 0.1 to 24.2/km^2^ with a mean of 2.5/km^2^. Wild colony densities were highest in sub‐Saharan Africa with an average of 6.8/km^2^. African *A. mellifera* swarm frequently and maintain smaller colonies (McNally & Schneider, 1996), which enables them to utilise a wider range of nesting cavities (Baum et al., 2005) and can even build nests in the open (Saunders et al., 2021). These life history traits will likely have a large effect on the spatial distribution of wild colonies in areas where the majority are of African descent. For instance, in the southern USA, wild Africanised honey bees (hybrids of *A. mellifera scutellata* and European subspecies) were more likely to occupy man‐made nesting cavities (Baum et al., 2008) and form colony aggregations (Baum et al., 2005) than European colonies living in the same area.

The high wild colony densities reported in Africa are also in keeping with the fact that honey and brood are commonly harvested from wild colonies by tribes in Central Africa (Crittenden, 2011; Kajobe & Roubik, 2006). Traditional beekeeping in Africa also relies on the colonisation of log hives by wild swarms (Gratzer et al., 2021). In addition, the greater honeyguide (*Indicator indicator*) has evolved a sophisticated mutualism with humans in the detection and predation of wild colonies (Spottiswoode et al., 2016; Wood et al., 2014). Wild honey bees are clearly an important part of human life in Africa and it is unlikely that these complex relationships would have evolved if wild colonies were rare.

Wild colony densities in Europe (average of 0.26/km^2^) were significantly lower than all other regions and were approximately 25 times lower than those reported in Latin America and Africa (Figure 3). These results probably reflect, in part, differences in the carrying capacity of temperate and tropical ecosystems. For instance, it is possible that temperate ecosystems do not produce enough nectar or pollen to support many wild colonies and that they naturally occur at lower densities in Europe (Kohl & Rutschmann, 2018). In a sub‐tropical prairie, Baum (2003) found that the nectar and pollen produced by plants within a 6.3 km^2^ study area could support between 407 and 3161 wild colonies, but the number of colonies located during an 11‐year survey rarely exceed 75 (12/km^2^; Baum et al., 2005), suggesting that wild colony density was limited by other factors. Here, we show that wild colony densities remained significantly lower in Europe, but not Northern America, after controlling for climate.

It is possible that human activities are partly responsible for the low densities reported in Europe. For instance, it has been suggested that natural nest sites in old trees are lacking in many parts of Europe due to historical changes in land use (Carreck, 2008; Kohl et al., 2023; Kohl & Rutschmann, 2018; Oleksa et al., 2013) and that wild colonies are affected by the high density of managed colonies in this region through competition for food and the transfer of exotic pests and maladaptive genetics (Requier et al., 2019). However, estimates from 3 national parks in the Southern Urals (Ilyasov et al., 2015) suggested that wild colonies are living in Russian bee forests at similar densities (0.3/km^2^) to those recorded in the late 1600s (Galton, 1971), prior to major land use change (Chorley, 1981) and the advent of modern beekeeping in Europe (Crane, 1999). Although the historical density of wild colonies is not known for other parts of Europe.

It is also possible that wild colony densities were underestimated in parts of Europe. Four European studies inferred densities over large areas (average of 111 km^2^) by only searching for wild colonies in a specific habitat or nesting site, such as trees on rural avenues (Oleksa et al., 2013), black woodpecker (*Dryocopus martius*) nests in forests (Kohl & Rutschmann, 2018; Kohl et al., 2022) and concrete ‘power poles’ in an agricultural landscape (Rutschmann et al., 2022). Therefore, density was probably underestimated because wild colonies present in other habitats or nest sites within the survey area would not have been located. Indeed, we found that regional differences in wild colony density were no longer significant after controlling for both climate and survey area.

### 5.2 The effect of survey area

We found that survey area had a strong negative correlation with wild colony density. This might be because a greater proportion of colonies are not detected when survey efforts are spread across larger areas (>50 km^2^). In contrast, high densities produced by small survey areas (<5 km^2^) might be the result of a biasing effect whereby surveys are made in small areas where wild colonies are known to be abundant and do not reflect the density of wild colonies across the wider area in which the colonies forage. For instance, Oldroyd et al. (1994) located 37 wild colonies in a small area (0.25 km^2^) of riparian woodland in Victoria, Australia and reported a density of 148/km^2^. However, the density would have been considerably lower if the survey area had been extended to include neighbouring habitats that were unsuitable for nesting (i.e. without trees), but in which the wild colonies were foraging. If the foraging area is taken as a circle of radius 2, 3 or 5 km then the actual area would be approximately 12.6, 28.3 and 78.5 km^2^, leading to densities of approximately 2.9, 1.3 and 0.5/km^2^. Of course, there may well have been additional wild colonies in these wider areas, so it is not possible to determine the actual densities. However, it is clear that colony density in a nest site aggregation provided by a restricted area of suitable nesting habitat can be much higher than a colony density relevant to the foraging area of those colonies. Similar aggregations can also occur in *Apis dorsata*, a species that has open nests, where many colonies nest close to each other under branches of a tree or on a cliff (Oldroyd et al., 2000).

### 5.3 The effect of climate and land use

Temperature and net primary productivity were both positively correlated with wild colony density, which probably reflects an increase in foraging activity and the temporal availability of floral resources. Wild *A. mellifera* colonies exhibit seasonal migration in tropical Africa and America, which is considered an adaptation to changes in the spatial distribution of floral resources as it allows them to forage throughout much of the year (McNally & Schneider, 1992). In regions with lower mean annual temperatures, colonies must survive longer winters during which floral resources are scarce or absent and when it is often too cold to forage. In these regions, wild colonies experience elevated mortality during the winter months (up to 80% of founder colonies; Seeley, 2017) and this likely has a large effect on their density the following spring.

Wild colony densities peaked at mean annual temperatures of 23°C (Figure 4), which is consistent with an optimum foraging temperature of approximately 20°C (Abou‐Shaara et al., 2017). At mean annual temperatures exceeding 25°C, honey bee colonies are exposed to temperatures that negatively affect foraging and other aspects of colony productivity (Abou‐Shaara et al., 2017). Under these conditions, wild colony densities are probably limited by rainfall, which has been shown to be an important factor in seasonally arid locations (Baum et al., 2008; Loper et al., 2006; Oldroyd et al., 1994). Here, we show that rainfall is a less important factor on a global scale, which is consistent with Jaffé et al. (2010) who found that wild colony densities correlated with temperature, but not precipitation, at 25 sites across *A. mellifera*'s native range.

Land use and net primary productivity (an index of vegetation) did not significantly affect density at the 41 sampled locations. Honey bees are generalists and can forage over long distances in a variety of habitats (Beekman & Ratnieks, 2000; Ricigliano et al., 2019; Samuelson et al., 2020) so it is likely that land use only affects wild colony density at small spatial scales. In our analysis, land use was generalised over large areas, so we were unlikely to detect small‐scale variation in wild colony density, which is arguably more ecologically relevant (Utaipanon, Holmes, et al., 2019).

### 5.4 Comparisons with FAO data on managed colonies

Approximately half of all managed honey bee colonies worldwide (45.3 million) are in Asia (FAO, 2021) and the majority of these colonies are assumed to be *A. mellifera* of European descent (Osterman et al., 2021). Therefore, it is likely that managed swarms frequently escape into the wild, but it seems that they are unable to form self‐sustaining wild populations, although this needs to be verified. Proposed explanations include competition with native honey bees (e.g. *A. cerana*; Manila‐Fajardo & Cleofas, 2003), effects of native honey bee parasites (e.g. *Tropilaelaps clareae*; Oldroyd & Nanork, 2009) and difficulty in regulating brood production in tropical regions with little variation in day length (Rinderer, 1988). Indeed, European honey bees are poorly adapted to tropical climates (Harrison & Hall, 1993) and did not establish large wild populations in tropical America before Africanised honey bees were introduced (Michener, 1975; Quezada‐Euán et al., 1996).

Europe has the second‐highest number of managed honey bee colonies worldwide at 25.4 million (FAO, 2021). This includes data from 33 countries with a total landmass of 21.7 km^2^ and equates to a density of 1.2 managed colonies/km^2^ which is over four times higher than the average wild colony density reported in Europe. This suggests that a smaller proportion of colonies are subject to natural selection in Europe and that beekeeper management plays a more prominent role in the survival of both managed and wild colonies. For instance, the widespread use of veterinary treatments by beekeepers in Europe might help keep levels of pests and disease low enough for both managed and wild colonies to survive (Thompson, 2012). However, there are probably hotspots in Europe where wild colonies outnumber managed (Requier et al., 2020), and there has been an increased emphasis on natural beekeeping in recent years (Neumann & Blacquiere, 2016) where, amongst other suggestions, beekeepers are encouraged not to treat their colonies with chemicals so that they can evolve a natural resistance to disease (Seeley, 2019). Therefore, natural selection probably still contributes to colony survival in Europe, but not to the same extent as in other regions like Africa where wild colonies are more numerous and commercial beekeeping is poorly developed (Dietemann et al., 2009; Gratzer et al., 2021).

### 5.5 Estimated number of wild *A. mellifera* colonies

Based on the mean regional densities in Figure 3 and the estimated area of habitable landmass in each region, we estimate that there are approximately 280 million wild *A. mellifera* colonies worldwide, which is more than double the number of managed colonies reported by the FAO in 2021 (102 million). This is a tentative estimate based on limited data and should be used with caution. However, the strong indication is that wild *A. mellifera* colonies outnumber managed colonies in most regions, with the exception of Europe.

### 5.6 Areas for future research

Our study reveals several knowledge gaps regarding wild *A. mellifera* colony density which could be addressed by future research. An important area for future research concerns the limits to wild *A. mellifera*'s geographical range. In Europe, wild colonies are thought to occur as far north as 60 degrees latitude (Figure 2), which is consistent with the northernmost survey in our sample (Seeley & Chilcott, 2020). However, permanent beekeeping is practiced as far north as 68 degrees in Finland (Meyer‐Rochow, 2008) and it is possible that escaped swarms occur in northern settlements throughout the summer, but whether they survive the long winters at these latitudes is unknown. Similarly, little is known about wild colonies in the southernmost parts of *A. mellifera's* range, such as temperate South America. For instance, there is little information regarding wild colonies in the southern half of Argentina (below Buenos Aires), where the majority are of European descent (Rinderer et al., 1993). This also applies to much of Asia, where wild *A. mellifera* colonies are thought to be absent (Oldroyd & Nanork, 2009).

Analyses of wild colony density and numbers on smaller spatial scales, and possibly incorporating the effects of land use, might help identify hotspots in native regions where wild colonies outnumber managed (Requier et al., 2020). This would have implications for the conservation of native subspecies, given that wild colonies in these areas might represent local ecotypes and an important source of genetic diversity (Requier et al., 2019). For instance, wild colonies in Ireland are considered to be pure *A. mellifera mellifera* (Browne et al., 2020; Hasset et al., 2018), the subspecies native to Northern Europe. Although, managed colonies in Ireland are also mainly *A. mellifera mellifera* (NIHBS, 2021). The degree to which wild colonies are genetically distinct or significantly more native than managed colonies in other parts of Europe is an important topic for future research but is beyond the scope of this review.

## 6 CONCLUSION

In French, the honey bee (*A. mellifera*) is called ‘l'abeille domestique’, the domestic bee and in many countries, presumably including France, it is seen primarily as a bee that lives under human management in hives. However, our study clearly shows that the honey bee is also ‘une abeille sauvage’, that is living wild in unmanaged colonies (Seeley, 2019). Indeed, our results indicate that wild colonies outnumber managed colonies, although not in Europe. The realisation of this important fact should have significant consequences on how we view the honey bee. For example, the vast numbers of wild colonies, not to mention the approximately 102 million managed colonies, surely mean that the word endangered, which is frequently used by the media in the context of the honey bee, is far from accurate even though beekeepers, especially those in North America and Europe, have faced increased challenges in maintaining healthy live colonies in recent decades (Genersch, 2010). On a positive note, it also shows that in surviving its challenges the honey bee will be aided by natural selection on wild colonies in many locations. This is shown, for example, by wild colony surveys in New York States's Arnot Forest several decades apart using the bee‐lining method (Seeley, 2007; Visscher & Seeley, 1982), which showed the same colony density before and after the arrival of *Varroa* mites which are now found in the wild colonies (Seeley, 2019).

## AUTHOR CONTRIBUTIONS

**Oliver D. Visick:** Conceptualization (equal); data curation (lead); formal analysis (lead); visualization (lead); writing – original draft (lead). **Francis L. W. Ratnieks:** Conceptualization (equal); funding acquisition (lead); supervision (lead); writing – original draft (supporting); writing – review and editing (lead).

## FUNDING INFORMATION

This work was supported by the C.B. Dennis British Beekeepers' Research Trust.

## CONFLICT OF INTEREST STATEMENT

The authors declare that they have no known competing financial interests or personal relationships that could have appeared to influence the work reported in this paper.

## Supporting information


Table S1

Table S2

## Data Availability

The data that supports the findings of this study are available in the [Link], [Link] of this article. Data including R script and QGIS Shapefiles will also be uploaded to Dryad upon acceptance.
