# Supplementary material for: Density of wild honey bee, Apis mellifera, colonies worldwide
Source: Ecol Evol. 2023 Oct 11;13(10):e10609. doi: 10.1002/ece3.10609 (PMC10568204; doi:10.1002/ece3.10609)
Supplement: Supplementary file 1 — Table S1 [file ECE3-13-e10609-s001.docx]

**Table S1. Sample of 40 locations worldwide used for analysis.** Densities are presented in ascending order.

| **Region** | **Location** | **Land use** | **Mean annual temperature (°C)** | **Mean monthly precipitation (mm)** | **Net primary productivity**  **(gC/m^3^/day)** | **Main survey method** | **No. colonies** | **Survey area (km^2^)** | **Colonies/km^2^** | **reference** |
| --- | --- | --- | --- | --- | --- | --- | --- | --- | --- | --- |
| Europe | Northern Poland | Disturbed | 8.22 | 52.35 | 1.33 | Direct search | 45 | 458.1 | 0.1 | Oleksa *et al*. (2013) |
| Europe | Cawdor Wood | Mixed | 7.15 | 95.16 | 1.27 | Bee-lining | 4 | 31.1 | 0.13 | Seeley & Chilcott (2020) |
| Europe | Hainich NP | Undisturbed | 8.49 | 49.56 | 1.43 | Bee-lining | 9 | 49.5 | 0.18 | Kohl & Rutschmann (2018) |
| Europe | Xinzo de Limia | Disturbed | 11.26 | 91.27 | 2.22 | Direct search | 26 | 136 | 0.19 | Rutschmann *et al*. (2022) |
| Europe | Swabian Alb | Undisturbed | 8.91 | 64.77 | 1.64 | Direct search | 20 | 97.2 | 0.21 | Kohl *et al*. (2022) |
| Northern America | Santa Cruz Island | Undisturbed | 14.81 | 28.84 | 1.86 | Bee-lining | 58 | 230 | 0.25 | Wenner (1989) |
| Europe | Coburg & Lichtenfels | Undisturbed | 8.61 | 48.83 | 1.64 | Direct search | 19 | 59.1 | 0.31 | Kohl *et al*. (2022) |
| Europe | Morozov estate | Undisturbed | 5 | 49.68 | 1.21 | Colony records | 75 | 180.9 | 0.41 | Galton (1971) |
| Northern America | Tucson | Disturbed | 19.92 | 27.52 | 0.3 | Colony records | 645 | 924.1 | 0.7 | Baum *et al*. (2008) |
| Northern America | Shindagin Hollow State Forest | Undisturbed | 8.23 | 81.22 | 1.66 | Bee-lining | 5 | 5.2 | 0.96 | Radcliffe and Seeley (2018) |
| Northern America | Arnot Forest | Undisturbed | 8.31 | 77.89 | 1.64 | Bee-lining | 9 | 8.5 | 1.06 | Seeley *et al*. (2015) |
| Africa | Cape Point | Undisturbed | 16.28 | 52.18 | 1.87 | Bee-lining | 59 | 50.4 | 1.17 | Tribe *et al*. (2017) |
| Oceania | South Australia | Undisturbed | 15.61 | 40.42 | 1.47 | Direct search | 78 | 58.2 | 1.34 | Paton (unpublished) |
| Latin America | Panama Canal | Disturbed | 26.08 | 198.51 | 2.22 | Colony records | 78 | 50 | 1.56 | Boreham & Roubik (1987) |
| Europe | Belgrade | Disturbed | 12.53 | 53.71 | 1.12 | Colony records | 460 | 233.3 | 1.97 | Bila Dubaić *et al*. (2021) |
| Northern America | Oswego | Disturbed | 8.7 | 83.99 | 1.79 | Local knowledge | 11 | 4.2 | 2.62 | Morse *et al*. (1990) |
| Oceania | Puckapunyal N. & S. | Undisturbed | 14.26 | 55.5 | 1.71 | Genetic markers | 12 | 4.5 | 2.67 | Arundel *et al*. (2014) |
| Oceania | Currawarna | Disturbed | 16.57 | 38.92 | 1.01 | Genetic markers | 236 | 86.48 | 2.73 | Utaipanon *et al*. (2019b) |
| Latin America | Belén | Disturbed | 26.81 | 152.56 | 2.22 | Bee-lining | 38 | 12.6 | 3.02 | Danka *et al*. (1994) |
| Oceania | Grong Grong | Disturbed | 16.06 | 43.49 | 1.13 | Genetic markers | 265 | 86.48 | 3.06 | Utaipanon *et al*. (2019b) |
| Oceania | Marysville & Eildon | Mixed | 12 | 90.68 | 3.48 | Genetic markers | 28 | 9 | 3.11 | Arundel *et al*. (2014) |
| Oceania | Puckapunyal E. & Dookie | Mixed | 15.26 | 49.49 | 1.59 | Genetic markers | 32 | 9 | 3.56 | Arundel *et al*. (2014) |
| Northern America | Cottonwood | Undisturbed | 15.33 | 36.94 | 0.74 | Direct search | 12 | 3.1 | 3.87 | Taber (1979) |
| Africa | Okavango | Undisturbed | 23.35 | 38.79 | 0.7 | Direct search | 81 | 19.3 | 4.2 | McNally & Schneider (1996) |
| Africa | Al-Faw | Disturbed | 28.95 | 36.4 | 0.08 | Genetic markers | 19 | 4.5 | 4.22 | Jaffe *et al*. (2010) |
| Latin America | Goiás & Mato Grosso | Mixed | 24.07 | 126.76 | 1.39 | Direct search | 8 | 1.8 | 4.44 | Kerr (1971) |
| Northern America | Welder Wildlife Refuge | Undisturbed | 22.19 | 64.94 | 1.07 | Direct search | 28 | 5.1 | 5.49 | Rangel *et al*. (2016) |
| Africa | Tswalu game reserve | Undisturbed | 20.05 | 26.05 | 0.31 | Genetic markers | 15 | 2.5 | 5.8 | Jaffe *et al*. (2010) |
| Latin America | Tapachula | Disturbed | 25.35 | 155.86 | 3.29 | Local knowledge | 27 | 4.1 | 6.59 | Ratnieks *et al*. (1991) |
| Oceania | Barrington Tops | Mixed | 15.87 | 78.73 | 3.95 | Genetic markers | 77 | 10 | 7.7 | Hinson *et al*. (2015) |
| Africa | Gauteng province | Undisturbed | 17.23 | 53.99 | 1.37 | Genetic markers | 64 | 7.5 | 8.53 | Jaffe *et al*. (2010) |
| Oceania | Weddin Shire | Mixed | 16.76 | 45.05 | 1.24 | Genetic markers | 95 | 10 | 9.5 | Hinson *et al*. (2015) |
| Africa | Pietermaritzburg | Undisturbed | 17.67 | 75.14 | 3.21 | Genetic markers | 25 | 2.5 | 10 | Jaffe *et al*. (2010) |
| Africa | Jonkershoek | Undisturbed | 16.5 | 56.32 | 1.73 | Genetic markers | 26 | 2.5 | 10.4 | Jaffe *et al*. (2010) |
| Oceania | Adelaide | Mixed | 16 | 41.42 | 1.87 | Direct search | 81 | 7.65 | 10.59 | Williamson *et al*. (2022) |
| Oceania | Wimmera | Mixed | 16.19 | 27.39 | 0.93 | Genetic markers | 107 | 10 | 10.65 | Hinson *et al*. (2015) |
| Africa | Bwindi Impenetrable NP | Undisturbed | 22.17 | 86.22 | 3.23 | Local knowledge | 20 | 1.7 | 11.76 | Kajobe & Roubik (2006) |
| Latin America | Chiapas S. | Mixed | 25.66 | 164.79 | 3.14 | Genetic markers | 184 | 12.5 | 14.72 | Moritz *et al*. (2013) |
| Latin America | Yucatan | Undisturbed | 26.28 | 83.89 | 2.82 | Genetic markers | 79 | 5 | 15.8 | Moritz *et al*. (2013) |
| Latin America | Chiapas N. | Disturbed | 20.3 | 126.68 | 4.23 | Genetic markers | 228 | 12.5 | 18.24 | Moritz *et al*. (2013) |
| Africa | Lusaka | Undisturbed | 21.78 | 79.02 | 1.7 | Direct search | 29 | 1.2 | 24.17 | Coppinger *et al*. (2019) |
