# Supplementary material for: Density of wild honey bee, Apis mellifera, colonies worldwide
Source: Ecol Evol. 2023 Oct 11;13(10):e10609. doi: 10.1002/ece3.10609 (PMC10568204; doi:10.1002/ece3.10609)
Supplement: Supplementary file 2 — Table S2 [file ECE3-13-e10609-s002.docx]

**Table S2.** Managed honey bee colony numbers in 117 countries were FAO data on managed colonies are available. Managed colony numbers are the most recently reported by the FAO (mostly from 2021).

| **Region** | **Country** | **No. managed colonies** | **Year** | **FAO description** |
| --- | --- | --- | --- | --- |
| Africa | Algeria | 426,470 | 2021 | Estimated value |
| Africa | Angola | 1,187,290 | 2021 | Imputed value |
| Africa | Burundi | 199,390 | 2021 | Imputed value |
| Africa | Cameroon | 314,310 | 2021 | Imputed value |
| Africa | Central African Republic | 1,642,870 | 2021 | Estimated value |
| Africa | Chad | 124,870 | 2021 | Imputed value |
| Africa | Egypt | 749,150 | 2021 | Imputed value |
| Africa | Ethiopia | 7,105,880 | 2021 | Imputed value |
| Africa | Guinea | 65,210 | 2021 | Imputed value |
| Africa | Guinea-Bissau | 6,750 | 2021 | Imputed value |
| Africa | Kenya | 1,426,470 | 2021 | Official figure |
| Africa | Libya | 36,790 | 2021 | Imputed value |
| Africa | Madagascar | 135,720 | 2021 | Imputed value |
| Africa | Mali | 38,180 | 2021 | Imputed value |
| Africa | Morocco | 396,360 | 2021 | Imputed value |
| Africa | Mozambique | 47,200 | 2021 | Estimated value |
| Africa | Rwanda | 4,230 | 2021 | Estimated value |
| Africa | Senegal | 328,710 | 2021 | Imputed value |
| Africa | South Africa | 71,800 | 2021 | Estimated value |
| Africa | Sudan | 76,350 | 2021 | Imputed value |
| Africa | Tunisia | 669,040 | 2021 | Estimated value |
| Africa | Uganda | 81,600 | 2021 | Imputed value |
| Africa | Tanzania | 3,050,670 | 2021 | Imputed value |
| Africa | Zambia | 60,710 | 2021 | Estimated value |
| Asia | Armenia | 228,120 | 2021 | Imputed value |
| Asia | Azerbaijan | 625,300 | 2021 | Official figure |
| Asia | China | 9,216,660 | 2021 | Imputed value |
| Asia | Taiwan | 167,020 | 2021 | Official figure |
| Asia | Cyprus | 55,000 | 2021 | Official figure |
| Asia | Georgia | 228,500 | 2021 | Official figure |
| Asia | India | 12,848,200 | 2021 | Estimated value |
| Asia | Iran | 7,527,260 | 2021 | Estimated value |
| Asia | Israel | 121,000 | 2021 | Official figure |
| Asia | Japan | 197,210 | 2021 | Imputed value |
| Asia | Jordan | 46,800 | 2021 | Official figure |
| Asia | Kyrgyzstan | 149,130 | 2021 | Official figure |
| Asia | Lebanon | 383,070 | 2021 | Imputed value |
| Asia | Mongolia | 13,380 | 2021 | Official figure |
| Asia | Myanmar | 66,250 | 2021 | Estimated value |
| Asia | Oman | 154,220 | 2021 | Official figure |
| Asia | Pakistan | 378,350 | 2021 | Imputed value |
| Asia | Republic of Korea | 2,184,410 | 2021 | Estimated value |
| Asia | Syrian Arab Republic | 532,210 | 2021 | Official figure |
| Asia | Tajikistan | 242,410 | 2021 | Imputed value |
| Asia | Timor-Leste | 20,000 | 2021 | Imputed value |
| Asia | Turkey | 8,733,390 | 2021 | Official figure |
| Asia | Uzbekistan | 644,390 | 2021 | Official figure |
| Asia | Viet Nam | 271,270 | 2021 | Estimated value |
| Asia | West Bank | 64,360 | 2021 | Official figure |
| Asia | Yemen | 167,140 | 2021 | Imputed value |
| Europe | Albania | 393,640 | 2021 | Official figure |
| Europe | Austria | 456,000 | 2021 | Official figure |
| Europe | Belarus | 203,410 | 2021 | Imputed value |
| Europe | Belgium | 33,310 | 2017 | Imputed value |
| Europe | Bosnia and Herzegovina | 262,950 | 2021 | Official figure |
| Europe | Bulgaria | 838,000 | 2021 | Official figure |
| Europe | Croatia | 460,000 | 2021 | Official figure |
| Europe | Czech Republic | 661,000 | 2021 | Official figure |
| Europe | Estonia | 50,000 | 2021 | Official figure |
| Europe | Finland | 86,000 | 2021 | Official figure |
| Europe | France | 1,808,000 | 2021 | Official figure |
| Europe | Germany | 982,000 | 2021 | Official figure |
| Europe | Greece | 2,183,000 | 2021 | Official figure |
| Europe | Hungary | 1,207,000 | 2021 | Official figure |
| Europe | Italy | 1,717,000 | 2021 | Official figure |
| Europe | Latvia | 104,000 | 2021 | Official figure |
| Europe | Lithuania | 209,190 | 2021 | Official figure |
| Europe | Luxembourg | 3,000 | 2021 | Official figure |
| Europe | Moldova | 184,900 | 2021 | Official figure |
| Europe | Montenegro | 71,930 | 2021 | Imputed value |
| Europe | Netherlands | 78,000 | 1987 | Estimated value |
| Europe | North Macedonia | 312,620 | 2021 | Official figure |
| Europe | Poland | 2,013,000 | 2021 | Official figure |
| Europe | Portugal | 758,000 | 2021 | Official figure |
| Europe | Romania | 2,353,000 | 2021 | Official figure |
| Europe | Russia | 2,889,690 | 2021 | Official figure |
| Europe | Serbia | 976,440 | 2021 | Official figure |
| Europe | Slovak Republic | 344,000 | 2021 | Official figure |
| Europe | Slovenia | 213,000 | 2021 | Official figure |
| Europe | Spain | 2,953,000 | 2021 | Official figure |
| Europe | Sweden | 179,000 | 2021 | Official figure |
| Europe | Switzerland | 196,450 | 2021 | Official figure |
| Europe | United Kingdom | 191,000 | 1987 | Estimated value |
| Latin America | Argentina | 2,964,680 | 2021 | Imputed value |
| Latin America | Belize | 2,030 | 2021 | Imputed value |
| Latin America | Brazil | 1,030,450 | 2021 | Imputed value |
| Latin America | Chile | 409,350 | 2021 | Estimated value |
| Latin America | Colombia | 108,360 | 2021 | Estimated value |
| Latin America | Costa Rica | 39,830 | 2021 | Imputed value |
| Latin America | Cuba | 221,200 | 2021 | Official figure |
| Latin America | Dominican Republic | 95,490 | 2021 | Imputed value |
| Latin America | Ecuador | 47,520 | 2021 | Imputed value |
| Latin America | El Salvador | 216,140 | 2021 | Imputed value |
| Latin America | Guadeloupe | 800 | 2006 | Estimated value |
| Latin America | Guatemala | 85,150 | 2021 | Estimated value |
| Latin America | Guyana | 4,360 | 2021 | Imputed value |
| Latin America | Haiti | 27,250 | 2021 | Imputed value |
| Latin America | Honduras | 7,920 | 2021 | Imputed value |
| Latin America | Jamaica | 70,050 | 2021 | Imputed value |
| Latin America | Mexico | 2,226,050 | 2021 | Official figure |
| Latin America | Paraguay | 59,040 | 2021 | Imputed value |
| Latin America | Puerto Rico | 2,950 | 2021 | Estimated value |
| Latin America | Trinidad and Tobago | 8,100 | 2021 | Official figure |
| Latin America | Uruguay | 573,950 | 2021 | Imputed value |
| Latin America | Venezuela | 10,970 | 2021 | Estimated value |
| Northern America | Canada | 728,990 | 2021 | Estimated value |
| Northern America | United States of America | 2,696,000 | 2021 | Official figure |
| Oceania | Australia | 559,020 | 2021 | Imputed value |
| Oceania | Cook Islands | 50 | 2021 | Estimated value |
| Oceania | Fiji | 14,000 | 2021 | Official figure |
| Oceania | French Polynesia | 1,650 | 2021 | Estimated value |
| Oceania | New Caledonia | 11,610 | 2021 | Official figure |
| Oceania | New Zealand | 806,140 | 2021 | Official figure |
| Oceania | Niue | 820 | 2021 | Imputed value |
| Oceania | Samoa | 11,120 | 2021 | Imputed value |
| Oceania | Tonga | 940 | 2021 | Estimated value |
| Oceania | Tuvalu | 40 | 2021 | Imputed value |
